# Supplementary material for: A Critical View of the Application of the APEX Software (Aqueous Photochemistry of Environmentally-Occurring Xenobiotics) to Predict Photoreaction Kinetics in Surface Freshwaters
Source: Molecules. 2019 Dec 18;25(1):9. doi: 10.3390/molecules25010009 (PMC7017383; doi:10.3390/molecules25010009)
Supplement: Supplementary file 1 [file molecules-25-00009-s001.zip › 3.2.4_gcc-4.4.0.zip!/3.2.4_gcc-4.4.0/3.2.4_gcc-4.4.0/doc/octave/html/interpreter/Installation-Problems.html]

Installation Problems - Untitled


Up: Installation


---

### F.1 Installation Problems

This section contains a list of problems (and some apparent problems
that don't really mean anything is wrong) that may show up during
installation of Octave.

- On some SCO systems, `info` fails to compile if
  `HAVE_TERMIOS_H` is defined in config.h. Simply removing
  the definition from info/config.h should allow it to compile.- If `configure` finds `dlopen`, `dlsym`, `dlclose`,
    and `dlerror`, but not the header file dlfcn.h, you need to
    find the source for the header file and install it in the directory
    usr/include. This is reportedly a problem with Slackware 3.1.
    For Linux/GNU systems, the source for dlfcn.h is in the
    `ldso` package.- Building .oct files doesn't work.

      You should probably have a shared version of `libstdc++`. A patch
      is needed to build shared versions of version 2.7.2 of `libstdc++`
      on the HP-PA architecture. You can find the patch at
      ftp://ftp.cygnus.com/pub/g++/libg++-2.7.2-hppa-gcc-fix.- On some alpha systems there may be a problem with the `libdxml`
        library, resulting in floating point errors and/or segmentation faults in
        the linear algebra routines called by Octave. If you encounter such
        problems, then you should modify the configure script so that
        `SPECIAL_MATH_LIB` is not set to `-ldxml`.- On FreeBSD systems Octave may hang while initializing some internal
          constants. The fix appears to be to use

          ```
                    options      GPL_MATH_EMULATE
          ```

          rather than

          ```
                    options      MATH_EMULATE
          ```

          in the kernel configuration files (typically found in the directory
          /sys/i386/conf. After making this change, you'll need to rebuild
          the kernel, install it, and reboot.- If you encounter errors like

            ```
                      passing `void (*)()' as argument 2 of
                        `octave_set_signal_handler(int, void (*)(int))'
            ```

            or

            ```
                      warning: ANSI C++ prohibits conversion from `(int)'
                               to `(...)'
            ```

            while compiling sighandlers.cc, you may need to edit some files
            in the `gcc` include subdirectory to add proper prototypes for functions
            there. For example, Ultrix 4.2 needs proper declarations for the
            `signal` function and the `SIG_IGN` macro in the file
            signal.h.

            On some systems the `SIG_IGN` macro is defined to be something like
            this:

            ```
                      #define  SIG_IGN  (void (*)())1
            ```

            when it should really be something like:

            ```
                      #define  SIG_IGN  (void (*)(int))1
            ```

            to match the prototype declaration for the `signal` function. This
            change should also be made for the `SIG_DFL` and `SIG_ERR`
            symbols. It may be necessary to change the definitions in
            sys/signal.h as well.

            The `gcc` `fixincludes` and `fixproto` scripts should
            probably fix these problems when `gcc` installs its modified set of
            header files, but I don't think that's been done yet.

            **You should not change the files in** /usr/include. You
            can find the `gcc` include directory tree by running the command

            ```
                      gcc -print-libgcc-file-name
            ```

            The directory of `gcc` include files normally begins in the same directory
            that contains the file libgcc.a.- Some of the Fortran subroutines may fail to compile with older versions
              of the Sun Fortran compiler. If you get errors like

              ```
                        zgemm.f:
                        	zgemm:
                        warning: unexpected parent of complex expression subtree
                        zgemm.f, line 245: warning: unexpected parent of complex
                          expression subtree
                        warning: unexpected parent of complex expression subtree
                        zgemm.f, line 304: warning: unexpected parent of complex
                          expression subtree
                        warning: unexpected parent of complex expression subtree
                        zgemm.f, line 327: warning: unexpected parent of complex
                          expression subtree
                        pcc_binval: missing IR_CONV in complex op
                        make[2]: *** [zgemm.o] Error 1
              ```

              when compiling the Fortran subroutines in the libcruft
              subdirectory, you should either upgrade your compiler or try compiling
              with optimization turned off.- On NeXT systems, if you get errors like this:

                ```
                          /usr/tmp/cc007458.s:unknown:Undefined local
                                symbol LBB7656
                          /usr/tmp/cc007458.s:unknown:Undefined local
                                symbol LBE7656
                ```

                when compiling Array.cc and Matrix.cc, try recompiling
                these files without `-g`.- Some people have reported that calls to shell\_cmd and the pager do not
                  work on SunOS systems. This is apparently due to having
                  `G_HAVE_SYS_WAIT` defined to be 0 instead of 1 when compiling
                  `libg++`.- On NeXT systems, linking to libsys\_s.a may fail to resolve the
                    following functions

                    ```
                              _tcgetattr
                              _tcsetattr
                              _tcflow
                    ```

                    which are part of libposix.a. Unfortunately, linking Octave with
                    `-posix` results in the following undefined symbols.

                    ```
                              .destructors_used
                              .constructors_used
                              _objc_msgSend
                              _NXGetDefaultValue
                              _NXRegisterDefaults
                              .objc_class_name_NXStringTable
                              .objc_class_name_NXBundle
                    ```

                    One kluge around this problem is to extract termios.o from
                    libposix.a, put it in Octave's src directory, and add it
                    to the list of files to link together in the makefile. Suggestions for
                    better ways to solve this problem are welcome!- If Octave crashes immediately with a floating point exception, it is
                      likely that it is failing to initialize the IEEE floating point values
                      for infinity and NaN.

                      If your system actually does support IEEE arithmetic, you should be able
                      to fix this problem by modifying the function `octave_ieee_init` in
                      the file lo-ieee.cc to correctly initialize Octave's internal
                      infinity and NaN variables.

                      If your system does not support IEEE arithmetic but Octave's configure
                      script incorrectly determined that it does, you can work around the
                      problem by editing the file config.h to not define
                      `HAVE_ISINF`, `HAVE_FINITE`, and `HAVE_ISNAN`.

                      In any case, please report this as a bug since it might be possible to
                      modify Octave's configuration script to automatically determine the
                      proper thing to do.- If Octave is unable to find a header file because it is installed in a
                        location that is not normally searched by the compiler, you can add the
                        directory to the include search path by specifying (for example)
                        `CPPFLAGS=-I/some/nonstandard/directory` as an argument to
                        `configure`. Other variables that can be specified this way are
                        `CFLAGS`, `CXXFLAGS`, `FFLAGS`, and `LDFLAGS`.
                        Passing them as options to the configure script also records them in the
                        config.status file. By default, `CPPFLAGS` and
                        `LDFLAGS` are empty, `CFLAGS` and `CXXFLAGS` are set to
                        `"-g -O"` and `FFLAGS` is set to `"-O"`.
